# Resuscitative endovascular balloon occlusion of the aorta (REBOA) in interhospital trauma care: A case report enabled by real-time information coordination

**DOI:** 10.1097/MD.0000000000047840

**Published:** 2026-02-20

**Authors:** Haojian Deng, Junrui Li, Jun Ma, Xiaogang Li, Linhua Wei, Tao Gan

**Affiliations:** a Department of Emergency Medicine, Liuzhou People’s Hospital Affiliated with Guangxi Medical University, Liuzhou, China; b Liuzhou Emergency and Medical Rescue Talent Little Heights, Liuzhou, China; c Liuzhou Key Laboratory of Emergency and Critical Care Medical Research, Liuzhou, China; d Department of Emergency Medicine, Xiangya Hospital, Central South University, Changsha, China.

**Keywords:** case report, information coordination, interhospital transport, mobile healthcare, partial aortic occlusion

## Abstract

**Rationale:**

Resuscitative endovascular balloon occlusion of the aorta (REBOA) is a key skill for managing noncompressible torso hemorrhage, but its use in prehospital transport remains controversial due to potential ischemic complications. The feasibility of REBOA during complex interhospital transfers, particularly in remote settings, requires further illustration.

**Patient concerns:**

A 51-year-old male presented with hemorrhagic shock (blood pressure 44/22 mm Hg, heart rate 160 bpm, cold peripheries) following a road traffic accident causing an open pelvic fracture and right femoral shaft fracture.

**Diagnoses:**

Open pelvic fracture, right femoral shaft fracture, and traumatic hemorrhagic shock. Injury Severity Score was 17.

**Interventions:**

Partial REBOA was performed in an ambulance during interhospital transport, enabled by a regional real-time 5G-based medical information coordination system that facilitated dynamic ambulance rendezvous and remote consultation. A 14 mm ATLAS PTA balloon catheter (Bard) was advanced to zone III via a 7 Fr sheath (Terumo, Japan), with blood-pressure-guided partial occlusion.

**Outcomes:**

Following REBOA deployment, hemodynamics stabilized (blood pressure increased to 102/82 mm Hg), allowing safe transport and subsequent definitive surgical hemostasis. The patient survived without REBOA-related complications during the 6-month follow-up.

**Lessons:**

This case illustrates that, when supported by robust information coordination, REBOA may be feasibly and safely applied during complex interhospital transfers. It highlights the potential role of integrated telemedicine and coordination systems in extending advanced resuscitative care to challenging environments.

## 1. Introduction

Traumatic massive hemorrhage is an extremely dangerous issue in prehospital emergency medicine, particularly for hemorrhagic shock patients and patients with pelvic fractures, whose mortality rate can be as high as 60%.^[1]^ Resuscitative endovascular balloon occlusion of the aorta (REBOA) is a rescue procedure that temporarily occludes the aorta to augment proximal perfusion pressure and thereby ensure blood flow to vital organs such as the heart and brain and gain time for definitive hemostatic procedures.^[2]^ Although REBOA has been well established in hospital settings and controlled environments, safety and feasibility during interfacility transport are issues of ongoing debate.^[3]^

In China, the uneven distribution of medical resources, especially a lack of healthcare facilities in mountainous areas away from big cities, is one of the main obstacles for rapid transfer of high-risk trauma patients. However, the development of an information-based coordination system is an optimistic solution.^[4]^ Real-time sharing of imaging information, patient condition, and dynamic ambulance asset deployment on a 5G network enables trauma centers to seamlessly integrate prehospital care with hospital treatment.^[5]^ However, the use of REBOA in an ambulatory medical setting has certain challenges, such as geometric constraints, device stability, and operational precision. Up to now, there are no international or national reports on the use of REBOA under these particular circumstances.

**Figure 1.**
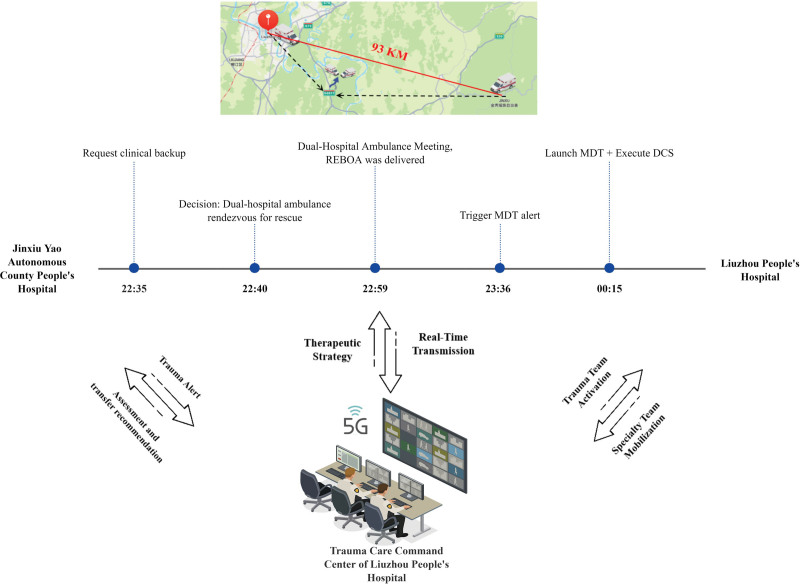
Real-time coordinated rescue timeline: dual-hospital ambulance rendezvous. DCS = damage control surgery, MDT = multidisciplinary team, REBOA = resuscitative endovascular balloon occlusion of the aorta.

This article addresses 2 key questions: the safety of partial REBOA during prolonged transport, and the feasibility of endovascular intervention in mobile medical environments by real-time coordination. It documents a successful case of REBOA implemented in an ambulance during patient transport in a remote mountainous area with the assistance of an information-sharing system on a regional scale. The study tries to offer a detailed feasibility report for clinical application of REBOA and investigate new models of trauma care facilitated by information technology (Fig. 1).

## 2. Case report

The patient was an 51-year-old male with no important medical history, admitted on November 02, 2022, to the People’s Hospital of Guangxi Jinxiu Yao Autonomous County following a multiple injury road accident. On admission, open pelvic fracture, right femoral shaft fracture, and hemorrhagic shock were diagnosed. He underwent initial stabilization in the form of manual fixation of the pelvis, fluid resuscitation, and blood transfusions. But definitive hemorrhage control surgery was not possible by the primary care facility. Four hours after the trauma, the patient’s blood pressure decreased to 60/30 mm Hg, necessitating the call for assistance and evacuation to a higher-level medical center.

When responding to a call for consultation, the Liuzhou People’s Hospital Trauma Center examined the patient and diagnosed his condition as hemorrhagic shock caused by fractures with active bleeding that required immediate blood transfusion and hemostatic treatment. With the 2 hospitals 93 km from each other, normally in about a 2-hour car ride, the command center executed the “Rendezvous Rescue” policy. The ambulances from both institutions were dispatched at the same time, proceeding in opposite directions and converging at a designated service point along Wu-Liu highway after 1.5 hours. The patient, upon reaching, presented with clinical features of shock like lethargy, heart rate of 160 beats/min, blood pressure of 44/22 mm Hg, and cold peripheries. The patient’s Injury Severity Score was rated to be 17 based on radiological assessments.

**Figure 2.**
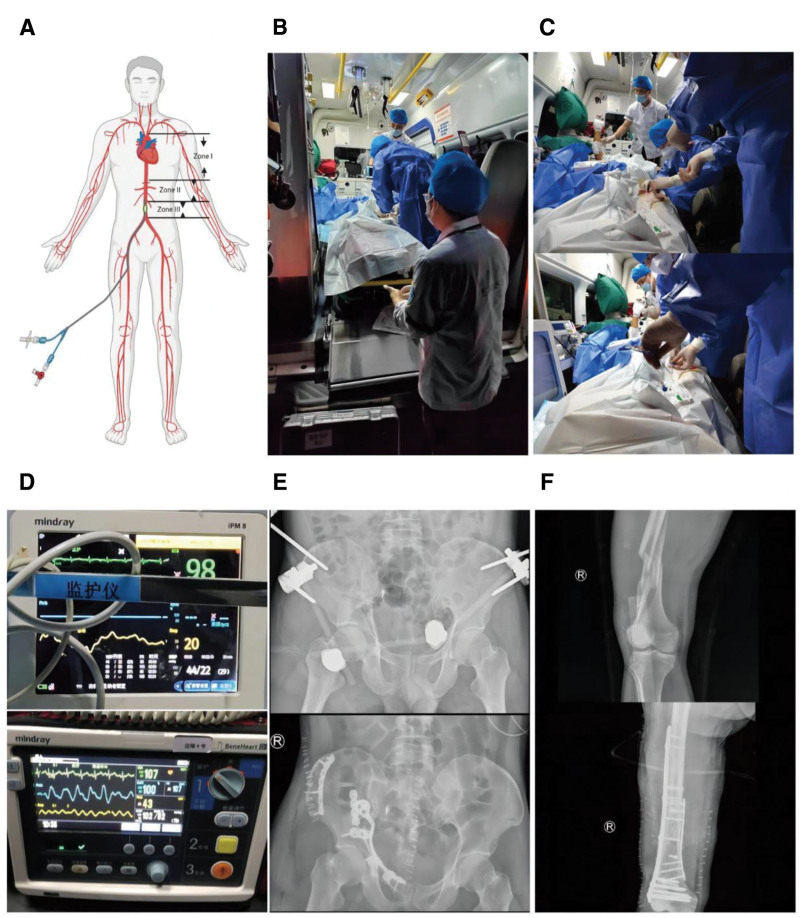
Inhospital and prehospital treatment process. (A) Aortic zones in REBOA conceptualization, (B, C) REBOA deployment by trauma resuscitation team in ambulance, (D) vital sign comparison: pre- versus post-REBOA, (E, F) preoperative and postoperative radiographs of pelvic and femoral internal fixation. REBOA = resuscitative endovascular balloon occlusion of the aorta.

Following real-time consultation, the trauma center directed the emergency response team to perform REBOA in the ambulance. Under guidance of a handheld ultrasound device, the left common femoral artery was punctured, and a 7 Fr RADIFOCUS Introducer II sheath (RS*A70K10SQ; Terumo, Tokyo, Japan) was placed. The procedure utilized a 14mm ATLAS^®^ PTA balloon dilatation catheter (Model: AT-75144/ 89095; Bard, Franklin Lakes), which was advanced through the sheath to zone III of the aorta. A blood pressure-guided partial REBOA strategy was employed, based on established clinical principles and our institutional protocol. The balloon was incrementally inflated to titrate the patient’s systolic blood pressure to a target range of 90 to 100 mm Hg. Distal perfusion was monitored by palpation of the contralateral femoral artery pulse and assessment of distal arterial flow using duplex ultrasound, thereby defining and maintaining the “partial” occlusion state. The duration of occlusion was dynamically adjusted based on these real-time hemodynamic and perfusion parameters. A dedicated nurse continuously monitored the procedure. Initial balloon inflation raised the patient’s blood pressure to 102/82 mm Hg. This titrated strategy was maintained during the remaining transport to the receiving hospital (Fig. 2A–D).

During the process of transfer, the hospital trauma green channel was initiated, making use of a 5G mobile terminal to track the patient’s vital signs and imaging data in real-time, thereby accelerating the admission process. The trauma center staff preprepared all resuscitation devices, medications, and blood transfusion products. A multidisciplinary team of experienced surgeons who specialize in trauma orthopedics and interventional radiology were available in standby within the treatment unit. On admission, the multidisciplinary team initiated external fixation of the pelvis and transcatheter arterial embolization of the internal iliac artery immediately (Fig. 2E, F). After achieving hemodynamic stability, definitive fracture repair was achieved by the trauma orthopedic team. Postoperatively, trauma-induced coagulopathy and pulmonary infection complications developed in the patient, but were successfully treated with integrative treatment. The patient recovered to pre-injury activity with no complication of REBOA. At 6-month telephone follow-up, he experienced acceptable ambulatory function recovery and actually thanked the concerted rescue effort with special mention of the timely REBOA placement during interhospital transport.

### 2.1. Ethical statement

This case report was reviewed and approved by the Medical Ethics Committee of Liuzhou People’s Hospital (Approval No. 2025(KY-E-06)). The committee confirmed that the manuscript contains no personally identifiable information of the patient and that the informed consent process was properly obtained and documented. Written informed consent for both the procedure and the publication of this case report was secured from the patient.

## 3. Discussion

This is the first reported case in Guangxi, China, demonstrating the successful use of REBOA by a prehospital mobile medical team. This was achieved via an inter-institutional real-time information collaboration system and led to cross-regional patient transfer. Historically, concerns have been raised that the risk of ischemic complications of REBOA might outweigh its temporary hemostatic benefit, especially when the duration of occlusion is longer than the known periods of safety.^[6]^ Nevertheless, this case demonstrates the effective use of REBOA during the interhospital transport of a patient with traumatic hemorrhagic shock using an information collaboration system. This case presents a detailed clinical illustration that adds to the ongoing discussion regarding the use of REBOA in acute practice, particularly in complex transfer scenarios.

The central debate revolves around achieving a balance between hemodynamic stability and risk of ischemia. The primary objective in the development of REBOA was to control noncompressible truncal hemorrhages. However, with advances in research, the disastrous complications of REBOA have become topics of equal significance. Previous research has shown that the complication rate of zone III REBOA is far lower than zone I. Yet, prolonged transport times still pose the threat of irreversible injury.^[7]^

Here, the patient’s bleeding from pelvic fracture was confined to zone III, and the total transport time was stringently maintained at 80 minutes, within the recommended limit. Compliant balloon catheters along with an intermittent occlusion method were employed, in which 30 minutes of occlusion alternated with 10 minutes of release. The method allowed continuous proximal perfusion with a reduction in ischemic stress in the lower limbs. This was in accordance with the new hypothesis of “partial REBOA,” where dynamically changing the degree of occlusion might be the best risk-reducing strategy. Application of REBOA gave the patient invaluable additional time to intervene further.^[8,9]^ Interestingly, REBOA increased afterload, stabilized hemodynamics, and preserved sufficient cerebral and cardiac perfusion. In this case, the observed hemodynamic stabilization was a pronounced effect. This observation suggests that the physiological benefits of REBOA may extend beyond direct hemorrhage control in specific scenarios, though generalizability is limited by the single-case nature of this report.

The geographic constraints of the mountainous regions of China’s Guangxi Province and the absence of rural healthcare facilities are major obstacles to trauma care. The journey road from Jinxiu Yao Autonomous County to Liuzhou City must travel through the mountainous bends of Dayaoshan, worse with poor road conditions, notably at night. Even though the 93-km distance of the journey is short, the journey would last longer than 2 hours due to the underdeveloped highway network. In these conditions, the regional information sharing system has achieved 3 major breakthroughs: Real-time decision support: based on the 5G network’s high-speed, low-latency characteristic, real-time transfer of high-definition CT imaging was achieved and provided trauma experts with precise diagnostic evidence and reduced misdiagnosis possibility. Concurrently, seamless integration of information from the hospital and ambulance enabled continuous transmission of vital signs and real-time in-field imaging. This integration allowed prehospital resource and team preparation to occur, subsequently reducing emergency response times significantly. Dynamic resource allocation: the command center utilized a “bidirectional dispatch” strategy for ambulances from both locations, reducing rendezvous time to 1.5 hours – a 40% improvement in transfer efficiency compared to traditional techniques. Comprehensive quality control: portable ultrasound-guided puncture and Bluetooth imaging were used to verify balloon position, minimizing procedural error to an absolute minimum.

Within the prehospital setting, the deployment of REBOA is also additionally challenged by key factors such as space limitations, device performance, and ensuring asepsis. For the purposes of safe and effective deployment, our team employed an encompassing strategy that combined anatomical surface landmarks with real-time ultrasound guidance to ensure accurate localization. Specifically, the midpoint of the umbilicus to pubic symphysis was used as the primary anatomical landmark with accurate placement of the balloon at the desired site. A single nurse constantly monitored distal pulses and balloon pressure in real-time so that occlusion strategy could be adjusted immediately. Intensive training on REBOA catheter placement was given to all members of the trauma resuscitation team, with over 20 simulated practice exercises, to ensure technical competence and coordinated team effort. The operation took 8 minutes to complete successfully, with minimal complications. Moreover, the research group was successful in establishing a new standardized management system for aortic balloon occlusion devices, prepackaged combined occlusion kits for immediate deployment, among others. With the implementation of a “grab-and-go” response model and using trained personnel, preparation time for prehospital REBOA was <2 minutes, a significant improvement in operational efficiency. The innovations and strategies described herein may serve as a valuable reference for teams considering or planning REBOA deployment in challenging prehospital settings (Fig. S1, Supplemental Digital Content, https://links.lww.com/MD/R449).

Despite the successful outcome of this case, several challenges persist that require attention: The absence of a rapid infusion system for universal blood products may impede the efficiency of resuscitation in patients experiencing severe shock. The inadequate aseptic conditions in mobile environments necessitate the implementation of more stringent disinfection protocols for ambulances and surgical procedures. The scarcity of large-scale and longitudinal data underscores the necessity for future research integrating artificial intelligence and portable extracorporeal circulation devices, with the aim of further expanding the application boundaries of REBOA in complex scenarios. These challenges highlight the need for ongoing innovation and optimization in prehospital emergency care.

This case illustrates that the implementation of REBOA, when supported by an information collaborative system, can be effectively and safely executed in mountainous interhospital transfer contexts. The success of this approach is contingent upon the integration of dynamic resource allocation, precise technical execution, and multidisciplinary collaboration. Consequently, this experience may offer a replicable model and highlights innovative approaches for trauma care in similar resource-constrained environments.

## Acknowledgments

The authors extend their gratitude to all members of the emergency response, trauma, and multidisciplinary teams involved in the prehospital and inhospital care of this patient. We also thank the colleagues from relevant departments for their clinical support.

## Author contributions

**Conceptualization:** Haojian Deng, Junrui Li, Xiaogang Li, Linhua Wei, Tao Gan.

**Data curation:** Haojian Deng, Junrui Li, Linhua Wei.

**Formal analysis:** Haojian Deng, Linhua Wei.

**Funding acquisition:** Tao Gan.

**Investigation:** Haojian Deng, Linhua Wei.

**Methodology:** Linhua Wei.

**Project administration:** Xiaogang Li, Tao Gan.

**Resources:** Tao Gan.

**Supervision:** Tao Gan.

**Validation:** Xiaogang Li, Tao Gan.

**Visualization:** Haojian Deng.

**Writing – original draft:** Haojian Deng, Junrui Li.

**Writing – review & editing:** Junrui Li, Jun Ma, Xiaogang Li, Linhua Wei, Tao Gan.

## Supplementary Material
