# Supplementary figures and images for: Resuscitative endovascular balloon occlusion of the aorta (REBOA) in interhospital trauma care: A case report enabled by real-time information coordination
Source: Medicine (Baltimore). 2026 Feb 20;105(8):e47840. doi: 10.1097/MD.0000000000047840 (PMC12928857; doi:10.1097/MD.0000000000047840)

****Supplementary Figure S1:****


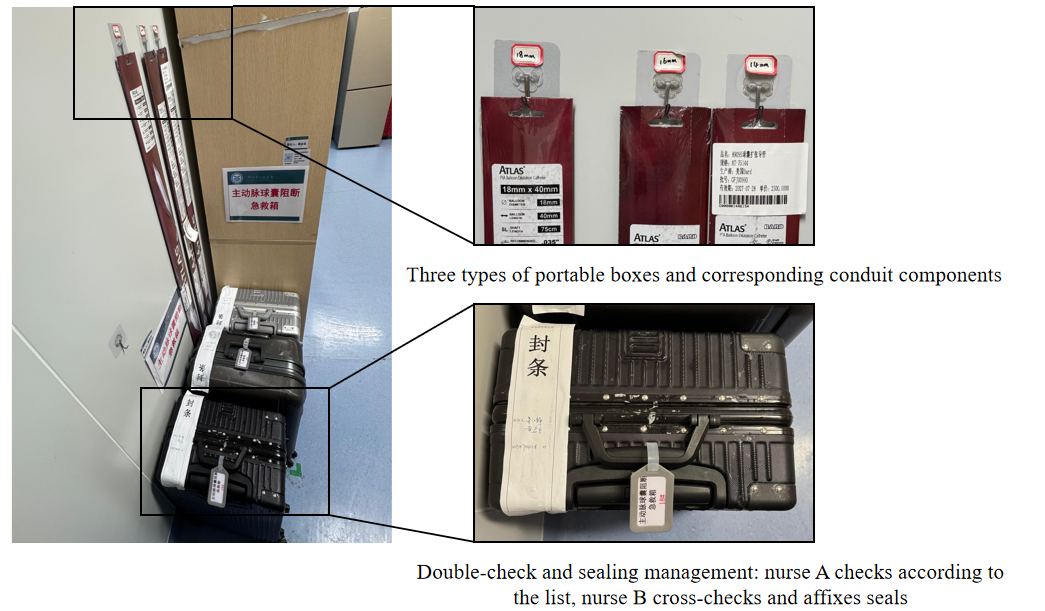


Fig S1. Dual-Verification Protocol for REBOA Portable Kits

Supplement: Supplementary file 1 [file medi-105-e47840-s001.docx]
